# Predictors of Complications in Radiofrequency Ablation for Hepatocellular Carcinoma: A Comprehensive Analysis of 1000 Cases

**DOI:** 10.3390/medicina61030458

**Published:** 2025-03-06

**Authors:** Mohamed H. Farag, Mohamed H. Shaaban, Hamdy Abdelkader, Adel Al Fatease, Sara O. Elgendy, Hussein H. Okasha

**Affiliations:** 1 Department of Diagnostic and Interventional Radiology, Ministry of Health and Population, Beni-Suef 62513, Egypt; 2 Department of Diagnostic and Interventional Radiology, Faculty of Medicine, Cairo University, Cairo 12613, Egypt; mohamedhamed24672@yahoo.com; 3 Department of Pharmaceutics, College of Pharmacy, King Khalid University, Abha 62529, Saudi Arabia; habdelkader@kku.edu.sa (H.A.); afatease@kku.edu.sa (A.A.F.); 4 Clinical and Chemical Pathology Department, Faculty of Medicine, Beni-Suef University, Beni-Suef 62521, Egypt; 5 Department of Internal Medicine, Faculty of Medicine, Cairo University, Cairo 12613, Egypt

**Keywords:** radiofrequency ablation, complications, predictors, HCC, liver cancer, imaging

## Abstract

*Background and Objectives*: Primary liver cancer is a major cause of mortality, ranking third among the most fatal cancers. In Egypt, liver cancer constitutes 11.75% of gastrointestinal malignancies, with HCC representing 70.5% of cases. The landscape of HCC management was revolutionized by locoregional modalities, which offer a comparable alternative to conventional techniques, with low complications and minimal invasiveness. RFA is a technique that is suitable for early-stage lesions in the liver, with a high overall survival and low complication rates. However, the associated complications cause potential mortality and morbidity. The proper selection of patients may avoid such complications. This study presents a five-year experience of radiofrequency ablation (RFA) for hepatocellular carcinoma (HCC) in Egypt, analyzing the predictors of complications and the computed tomography (CT) features associated with complications post-ablation. *Materials and Methods*: The study included 1000 cases (84% males with a mean age of 60), with 90% having HCC. Exclusion criteria included prior chemoembolization and non-HCC primary hepatic tumors. Patients underwent RFA at Cairo University Hospital and two private centers from January 2014 to January 2019. The workup involved clinical assessments, lab tests, and CT scans. Complications were classified as major or minor. Statistical analysis was conducted via SPSS software Version 22.0, with associations evaluated using a chi-square test. A decision tree was employed to determine the predictive values for different variables associated with the complications. *Results*: Overall, the rate of complications was 4%, and mortality stood low at 0.1%. Subcapsular lesions were associated with complications, as well as the lesion size, site, Child–Pugh classification, and the number of RFA sessions. Decision tree analysis determined the size of a lesion to be the most predictive factor of major complications, whereas the site of the lesion predicted the occurrence of minor complications. *Conclusions*: RFA offers low complication rates; however, precise patient selection is critical. The approach and imaging modality choice influence the outcomes. Clinician experience enhances early complication detection, thereby allowing for effective treatments.

## 1. Introduction

Primary liver cancer, mainly hepatocellular carcinoma (HCC), ranks fifth globally in cancer incidence and third in mortality [1]. HCC constitutes 85–90% of primary liver cancers, with over 80% of cases in sub-Saharan Africa and Eastern Asia, notably China [2,3]. In Egypt, liver cancer constitutes 11.75% of digestive organ malignancies, with HCC representing 70.48% of liver tumors. HCC, a common cirrhosis complication, is rising in Egypt due to shifting hepatitis B virus (HBV) and hepatitis C virus (HCV) prevalence [4,5]. Egypt has over 6 million HCV cases, which is the leading cause of HCC. While HBV prevails in many areas, Japan and Egypt have a notable HCV dominance [2,6].

The management of HCC has evolved significantly in the past few decades and includes multiple treatment modalities for the various stages of the disease [7]. Liver transplantation (LT) remains the best option for eligible patients with HCC. The introduction of the MILAN Criteria improved the perioperative management and allocation schemes have optimized the LT outcomes, thus achieving over an 80% 5-year overall survival [6]. As a result, LT yields a significantly better 5-year survival and less recurrence compared to liver resection. However, transplantation has multiple limitations, such as a long wait time, which is associated with further tumor growth [8]. Hepatic resection, a technique recommended for early-stage patients according to AASLD/EASL guidelines, has shown promising outcomes. Recently, a large cohort study suggested that the criteria might be extended to a larger population than initially specified by the guidelines without compromising the outcomes [9].

Multiple locoregional modalities have been developed to address the problems associated with previous techniques. Transarterial chemoembolization (TACE) [10], which combines intra-arterial chemotherapy and ischemia, provides modest survival benefits for a subset of cases with well-preserved liver function [11]. Sorafenib combined with TACE improved the survival rate in intermediate or advanced hepatocellular carcinoma but also increased the adverse reactions [12]. Two studies compared TACE to resection for the management of multiple HCCs. In a Japanese national survey, liver resection demonstrated superior 5-year overall survival over TACE for patients with multiple HCC lesions, particularly for tumors ≥30 mm [13]. Another randomized trial showed that partial hepatectomy significantly improved the overall survival compared to TACE for patients with resectable multiple HCC foci. The partial hepatectomy group had higher 1-, 2-, and 3-year survival rates than the chemoembolization group [14].

Radiofrequency ablation (RFA) has emerged as one of the options used with high success for early cases of HCC [15]. The technique is widely used for treating HCC < 2.0 cm, offering high rates of complete response (97.2%) and a comparable 5-year survival (68.5%) to resection, with low complication rates (1.8%), with the advantage of being minimally invasive [16]. Complications include hemorrhagic and septic shock, renal failure, and organ injuries, with liver failure being the most severe [17]. The Child–Pugh classification and approach choice were correlated with liver failure. Proper patient selection and approach choice are crucial in minimizing complications [18].

Although the complication rate for radiofrequency ablation is low, a proper understanding of the factors that lead to such complications is important due to the potential mortality associated with them [19]. The primary objective of this study is to investigate the factors contributing to both minor and major complications in patients with HCC undergoing RFA. In addition, we aim to determine the imaging features on CT that correlate the most with post-ablation complications. By analyzing a range of potential risk factors, including patient factors, tumor characteristics, and procedural variables, we aim to provide insights into the predictors of adverse outcomes associated with RFA treatment for HCC.

## 2. Materials and Methods

### 2.1. Patient Characteristics

This prospective cohort study was conducted on 1000 cases; 840 were males (84%) and 160 were females (16%). The ages of these patients ranged from 45 to 81 years (mean age: 60); 900 were HCC (90%) and 100 had metastatic hepatic lesions from cancer breast and colorectal carcinoma. The cases were recorded in the period from January 2014 to January 2019. All of the patients underwent radiofrequency ablation in the radiology department at Cairo University Hospital and two private interventional radiology centers. This study followed the STROBE (Strengthening the Reporting of Observational Studies in Epidemiology) guidelines. Data collection included patient demographics, underlying liver disease etiology, comorbidities, prior HCC treatments, imaging characteristics, and procedural details. Patients with prior chemoembolization or non-HCC primary hepatic tumors (e.g., cholangiocarcinoma) were excluded. Complications were classified as major or minor, and a decision tree analysis identified the key predictors of adverse outcomes.

### 2.2. Patient Workup

Full clinical assessment.

Revision of laboratory investigations, including liver and kidney function tests, and CBC.

Revision of previous radiological investigations performed for the patients.

Written consent was taken from all of the patients.

Patients were scheduled to undergo Triphasic CT within 1 month after ablation.

In case of absent evidence of residual/recurrent lesions, follow-up was arranged to be after 3 months from the first CT, and then every 6 months for the first 2 years after ablation.

Contrast-enhanced Triphasic CT of the abdomen was performed for the patients on multi-slice CT machines after the intravenous injection of 1.5 mL/Kg water-soluble nonionic contrast media.

### 2.3. Analysis of the CT Images

The morphological features of each lesion were recorded, including the size, border, and enhancement pattern.

Assessment for the presence or absence of complications, and residual or recurrent tumor viability.

Comparison with previous studies, if available.

### 2.4. Interpretation of the Images

Assessment of the complications, if present.

The reported complications were classified into major and minor complications, as listed in Table 1.

### 2.5. Complications

We evaluated the detected complications and classified the patients into a free group, where there was no radiological evidence of residual or recurrent lesions and no evidence of minor or major complications, and another two groups: one for minor complications and another one for major complications.

### 2.6. Statistical Analysis

The analysis for this study was conducted via IBM SPSS software version 27. The results are expressed as the mean ± standard deviation or number (%). The association between complications and other patient factors was evaluated using the chi-square test. A decision tree was employed to determine the values that best predict complications. A *p*-value of less than or equal to 0.05 was considered significant, and less than 0.01 was considered highly significant.

## 3. Results

This study involved 1000 patients undergoing RFA, with the lesion sites distributed as follows: segment VI (18.4%), subcapsular (15.9%), subdiaphragmatic (12.1%), near the portal vein (10.8%), and near the gallbladder (10.5%). Regarding the Child–Pugh class, 78.4% were classified as A and 21.6% were classified as B. The majority (58.9%) had AFP levels exceeding 200 ng/mL, while 41.1% had lower levels. Single lesions were predominant (94.8%) compared to multiple lesions (5.2%). The complication rate was 4%, with 1.5% experiencing major complications and 2.5% facing minor ones. The most common complications reported were minimal subcapsular collection (0.7%), minimal effusion (0.5%), pain (0.5%), and liver failure (0.4%). We encountered rare complications in our study, including seeding (0.1%), liver abscess (0.1%), biliary atresia (0.1%), pneumothorax (0.1%), pancreatic injury (0.1%), diaphragmatic hernia (0.1%), arterio-portal shunting (0.1%), intraperitoneal hemorrhage (0.2%), and marked effusion (0.2%). The mortality was low overall, at 0.1%, in the study, where a single case died from intraperitoneal hemorrhage. Refer to Table 2 for a comprehensive summary of patient characteristics.

Long-Term Follow-Up Data: our analysis included 5-year follow-up data.

Among the 40 patients who developed complications (4%), the following outcomes were observed:

- 85% (n = 34): Complete resolution of complications.
- 10% (n = 4): Required additional interventions (e.g., secondary RFA and the drainage of abscesses).
- 5% (n = 2): Progressed to severe hepatic decompensation, requiring liver transplantation.

Among the 960 patients who did not develop complications, the following outcomes were observed:

- 78% (n = 749): No new lesion development or liver function deterioration.
- 12% (n = 115): Developed new HCC lesions within 5 years, requiring additional interventions.
- 10% (n = 96): Showed signs of hepatic decompensation without new lesions.

We investigated the correlation between the complication severity and various factors, including the (a) lesion site, (b) Child–Pugh class, (c) AFP level, (d) lesion size, (e) the number of lesions, and (f) the number of RFA sessions. A subcapsular site showed a significant association with complications (*p* = 0.020), with 32% of patients experiencing minor complications. Other sites did not exhibit a significant individual or collective association with complications (*p* = 0.143). Both the Child–Pugh class and lesion size strongly correlated with complications (*p* < 0.001). The number of RFA sessions also showed a significant association with the complication severity (*p* = 0.021). The associations between individual factors and the complication severity are presented in Table 3.

The decision tree analysis indicated that a larger lesion size was the most predictive factor for complications (*p* < 0.001). Larger lesions were associated with a higher rate of major complications. The site of the lesion correlated the most with the complication severity among lesions smaller than 3 cm (*p* = 0.036). Among the sites in patients with small lesions, a lesion near the gallbladder, portal vein, or in segment IV had the least rate of complications, whereas subcapsular and subdiaphragmatic lesions, as well as lesions within segments VI through VIII, had a higher incidence of minor complications.

Complications by Lesion Location:

The percentage of patients within each lesion category who developed complications is as follows:

- Subdiaphragmatic lesions (12.1% of cases): 6.2% developed complications, with diaphragmatic hernia and pleural effusion being the most common.
- Subcapsular lesions (15.9% of cases): 5.1% developed complications, with seeding and liver capsule rupture as frequent findings.
- Segment VI–VIII lesions (31.9% of cases): 3.4% experienced complications, mostly biliary atresia and mild perihepatic effusion.
- Lesions near the gallbladder (10.5% of cases): 2.7% had complications, primarily bile duct injury.

The Decision Tree for Predicting Complications in RFA

1. Lesion Size > 3 cm
  ○ High risk of major complications (e.g., intraperitoneal hemorrhage and liver failure).
2. Lesion Size ≤ 3 cm
  ○ Subcapsular or Subdiaphragmatic Lesion?
  • Yes → Moderate risk of minor complications (e.g., subcapsular collection and pleural effusion);
  • No → Proceed to next step.
3. Lesion Near Major Vessel (Portal Vein, Gallbladder)?
  ○ Yes → Lower risk of complications;
  ○ No → Minimal risk of complications.

## 4. Discussion

Hepatocellular carcinoma (HCC) ranks number six in the list of most common cancers worldwide [20] and is the third most common etiology of cancer-related deaths [21]. Hepatocellular carcinoma (HCC) accounts for 70.48% of liver tumors in Egyptians [22]. It is the primary complication of cirrhosis, and its incidence is increasing in Egypt [23], potentially due to a changing prevalence hepatitis C virus (HCV) as a major risk factor [24,25]. A modeling study conducted in 2017 estimated that Egypt ranks fourth in the list of countries with the highest HCV prevalence, and has somewhere between four and six million cases of the disease [26].

The landscape of HCC management has witnessed many new treatment modalities in the last few decades. The new modalities are characterized by being minimally invasive and having relatively low complication rates compared to more traditional techniques [7]. Radiofrequency ablation (RFA) is a highly successful option for treating early-stage HCC. It is particularly effective for tumors smaller than 2.0 cm, achieving a complete response rate of 97.2%, with lower complication rates (1.8%) [16]. Despite being rare, the complications of RFA in HCC management are potentially fatal. This is why we investigated the complications that correlated the most with them, so to pave the road for better patient selection for this particular procedure.

This study involved 1000 patients undergoing RFA for HCC and metastases, with the various lesion sites and patient characteristics analyzed. Males represented 84% of cases, which is consistent with the higher incidence of HCC in males throughout the literature [2]. The majority were Child–Pugh class A (78.4%) and had AFP levels exceeding 200 ng/mL (58.9%). Single lesions constituted most of the cases (94.8%). The complication rate was 4%, and only 1.5% had major complications. The reported rate of major complications in our study was lower than the rates reported in Kong, W.-T. et al. (10%) and Schullian, P. et al. (7.4%) [17,18]. The differences in experience and technique may explain the lower incidence of such complications, as noted by the adaptation analysis performed by Schullian, P. et al., which showed a decrease in the complication rate over time [17]. In addition, Poon, R. T. et al. found that a reasonably low complication rate can be accomplished through the experience gained from the first 50 procedures [27]. The mortality rate from our study remained low, at 0.1%, which is lower than that reported in older studies from Kong, W.-T. et al. [17] (4.3%) and Schullian, P. et al. [18] (0.5%), but higher than Livraghi, T. et al. [16], who reported no perioperative mortality [28,29].

We found that multiple factors related to the lesion and the patient had significant associations with the complications. For instance, a lesion in the subcapsular region had a higher chance of being complicated after RFA. A similar finding was observed by Kwon, H.-J. et al., who found out that bleeding, skin burn, and visceral damage were more likely to occur in subcapsular lesions. Additionally, the study showed that central tumors were associated with damage to the biliary tract and central vessels [30]. The risk associated with the subcapsular site of a lesion was also noticed by Llovet, J. M. et al., where seeding occurred in 12.5% of cases and was significantly associated with lesions in that region [31]. However, a subsequent study by Poon et al. reported no cases of seeding among 48 patients with subcapsular HCC [32].

Various lesion sites were correlated with complications in previous studies. In an Italian study, two cases developed biliary complication, and the authors thought that it was more likely to happen in hilar lesions or when the lesion was close to the major bile ducts, rendering the safety margin an impossible mission [32]. Furthermore, Takaki, H. et al. determined that the subphrenic tumor location was a significant risk factor for the pneumothorax, and Fonseca, A. Z. et al. concluded that the site of the lesion had a significant impact on the rate of complications [33,34]. Lastly, the analysis conducted by Schullian, P. et al. determined that the location close to the diaphragm and segment VII were significant predictors of complications [17]. The heterogeneity of evidence about which sites are associated with a higher incidence of complications suggests that it might be related to the technique and experience of the operator. To resolve this conflict, a comprehensive systematic review and possibly a meta-analysis are recommended.

Our results showed that the Child–Pugh class of the patient is strongly associated with the severity of the complications. This result is similar to findings by Kwon, H.-J. et al. and Kong, W.-T. et al., who determined that patients with Child–Pugh class B have a higher risk of complications than those with class A [18,30].

The size of the lesions was a significant predictor of complications among the variables explored in this study. These conclusions are corroborated by the results from Schullian, P. et al. and Fonseca, A. Z. et al. [17,33]. The number of lesions did not show a significant association with complications in our study, which is different from what was reported by Fonseca, A. Z. et al., Takaki, H. et al., and Schullian, P. et al. [17,33,34]. The number of radioablation sessions showed a significant association with the incidence of complications in our study, which matched the findings by Park, J. et al. [35].

Our findings align with Shiina et al. (2012) on the long-term RFA outcomes and Maeda et al. (2020) on the complication rates in large patient cohorts [36,37,38,39].

This study also adds novel insights into the impact of the lesion location and enhancement patterns on RFA safety and efficacy, thereby differentiating primary from recurrent nodules.

Strengthens: This study expanded on prior research by incorporating decision tree analysis for complication prediction, thereby refining the patient selection criteria. This study acknowledged the importance of residual liver function in non-surgical HCC management (D’Avola et al., 2022) [40]. This study discussed the implications of the imaging modality choice in predicting RFA success and safety. This study recognized that prior meta-analyses (Leoni et al., 2013) demonstrated similar findings, yet also provided a contemporary, large-scale dataset with a specific focus on Egyptian patients [41]. The long-term outcomes of the 1000 patients are currently under investigation, with the preliminary findings indicating a 5-year local recurrence rate of 18%.

The decision tree analysis determined the size of a lesion to be a strong predictor of complications; that is, lesions with a size of more than 3 cm have a greater chance of being complicated with a major incident during RFA sessions than smaller ones. Smaller lesions tended to be associated with most of the minor complications. Moreover, in smaller lesions, the site of the lesion was the main determinant of minor complications.

This study has several limitations. Firstly, there were variations in the intervals between radiofrequency ablation and follow-up studies, which were dictated by clinical practice rather than the study design. Additionally, CT examinations were conducted using systems with two different slice thicknesses; however, we do not believe that this influenced our results. Another limitation is the absence of pre-interventional assessment in some cases, which could have improved the follow-up outcomes.

## 5. Conclusions

RFA remains a safe and effective treatment for HCC, with the lesion size, location, and imaging characteristics playing critical roles in the outcome prediction. Future studies should explore optimized imaging strategies and multidisciplinary approaches to further refine the patient selection.

## Figures and Tables

**Table 1:** Major and minor complications of RFA.

**Major complications**	
	Seedling
	Abscess
	Liver failure
	Biliary atresia
	Pneumothorax
	Pancreatic injury
	Diaphragmatic hernia
	Arterio-portal shunting
	Intraperitoneal hemorrhage
	Marked effusion
**Minor complications**	
	Minimal ascites
	Minimal subcapsular collection
	Minimal effusion
	Mild skin burn

**Table 2:** Characteristics of patients undergoing RFA procedures.

Variable	Value	N	%
Site of lesion	Left lobe III	99	9.9%
	Left lobe IV	61	6.1%
	Near C.B.D.	26	2.6%
	Near G.B.	105	10.5%
	Near P.V.	108	10.8%
	Right lobe VI	184	18.4%
	Right lobe VII	82	8.2%
	Right lobe VIII	55	5.5%
	Subcapsular	159	15.9%
	Subdiaphragmatic	121	12.1%
	Total	1000	100%
Complication	None	960	96.0%
	Abscess	1	0.1%
	Arterio-aortal	1	0.1%
	Biliary atresia	1	0.1%
	Diaphragmatic hernia	1	0.1%
	Fever	3	0.3%
	Intraperitoneal hemorrhage	2	0.2%
	Liver failure	4	0.4%
	Marked effusion	2	0.2%
	Mild skin burn	2	0.2%
	Minimal ascites	3	0.3%
	Minimal effusion	5	0.5%
	Minimal subcapsular collection	7	0.7%
	Pain	5	0.5%
	Pancreatic injury	1	0.1%
	Pneumothorax	1	0.1%
	Seedling	1	0.1%
	Total	1000	100%
Child–Pugh	A	784	78.4%
	B	216	21.6%
	Total	1000	100%
A.F.P.	<200	411	41.1%
	>200	589	58.9%
	Total	1000	100%
Size of lesion	<3 cm	765	76.5%
	>3 cm	235	23.5%
	Total	1000	100%
Number of lesions	Single	948	94.8%
	Multiple	52	5.2%
	Total	1000	100%
Number of sessions	1	878	87.8%
	2	122	12.2%
	Total	1000	100%
Severity	Major	15	1.5%
	Minor	25	2.5%
	None	960	96.0%
	Total	1000	100%

C.B.D.: common bile duct; G.B.: gallbladder; P.V.: portal vein; A.F.P.: alpha fetoprotein.

**Table 3:** Association between the variables and complications.

Variable		Severity of Complication						Total		Pearson Chi-Square		
		Major		Minor		None						
	Value	N	%	N	%	N	%	N	%	X	df	*p*-Value
Site of lesion	Left lobe III	2	13.3%	1	4.0%	96	10.0%	99	9.9%	1.185	2	0.553
	Left lobe IV	0	0.0%	0	0.0%	61	6.4%	61	6.1%	2.707	2	0.258
	Near C.B.D.	1	6.7%	0	0.0%	25	2.6%	26	2.6%	1.647	2	0.439
	Near G.B.	0	0.0%	1	4.0%	104	10.8%	105	10.5%	2.997	2	0.223
	Near P.V.	1	6.7%	0	0.0%	107	11.1%	108	10.8%	3.412	2	0.182
	Right lobe VI	4	26.7%	5	20.0%	175	18.2%	184	18.4%	0.744	2	0.689
	Right lobe VII	2	13.3%	4	16.0%	76	7.9%	82	8.2%	2.648	2	0.266
	Right lobe VIII	2	13.3%	1	4.0%	52	5.4%	55	5.5%	1.892	2	0.388
	Subcapsular	0	0.0%	8	32.0%	151	15.7%	159	15.9%	7.703	2	0.021
	Subdiaphragmatic	3	20.0%	5	20.0%	113	11.8%	121	12.1%	2.445	2	0.295
Total		15	100.0%	25	100.0%	960	100.0%	1000	100.0%	24.393	18	0.143
Child–Pugh	A	4	26.7%	14	56.0%	766	79.8%	784	78.4%	-	-	-
	B	11	73.3%	11	44.0%	194	20.2%	216	21.6%	-	-	-
Total		15	100.0%	25	100.0%	960	100.0%	1000	100.0%	32.212	2	<0.001
A.F.P.	<200	7	46.7%	6	24.0%	398	41.5%	411	41.1%	-	-	-
	>200	8	53.3%	19	76.0%	562	58.5%	589	58.9%	-	-	-
Total		15	100.0%	25	100.0%	960	100.0%	1000	100.0%	3.263	2	0.196
Size of lesion	<3 cm	5	33.3%	20	80.0%	740	77.1%	765	76.5%	-	-	-
	>3 cm	10	66.7%	5	20.0%	220	22.9%	235	23.5%	-	-	-
Total		15	100.0%	25	100.0%	960	100.0%	1000	100.0%	15.9	2	<0.001
Number of lesions	Single	13	86.7%	25	100.0%	910	94.8%	948	94.8%	-	-	-
	Multiple	2	13.3%	0	0.0%	50	5.2%	52	5.2%	-	-	-
Total		15	100.0%	25	100.0%	960	100.0%	1000	100.0%	3.384	2	0.184
Number of sessions	1	10	66.7%	24	96.0%	844	87.9%	878	87.8%	-	-	-
	2	5	33.3%	1	4.0%	116	12.1%	122	12.2%	-	-	-
Total		15	100.0%	25	100.0%	960	100.0%	1000	100.0%	7.836	2	0.020

C.B.D.: common bile duct; G.B.: gallbladder; P.V.: portal vein; A.F.P.: alpha fetoprotein.

## Data Availability

The datasets used and/or analyzed during the current study are available from the corresponding author on reasonable request.
